# Effects of Discarded Masks on the Offshore Microorganisms during the COVID-19 Pandemic

**DOI:** 10.3390/toxics10080426

**Published:** 2022-07-28

**Authors:** Jinlan Liao, Shouping Ji, Yulang Chi

**Affiliations:** 1 School of Advanced Manufacturing, Fuzhou University, Quanzhou 362200, China; 208527256@fzu.edu.cn; 2 College of Oceanology and Food Science, Quanzhou Normal University, Quanzhou 362000, China

**Keywords:** COVID-19 pandemic, microplastics, discarded masks, 16S rRNA gene sequencing

## Abstract

Numerous disposable plastic masks had been produced and used for preventing the worldwide COVID-19 pandemic effectively. Discarded masks are a potential source of microplastic pollution in marine ecosystems. The effect of discarded masks on offshore microorganisms is still unclear. Herein, we profiled the interaction between the microplastics released by discarded masks and marine microbes. The effects of mask quantity, time, and environment on the microplastic-related communities were determined. We characterized the bacterial communities of each group using 16S rRNA gene sequencing and metagenomic sequencing and correlated the community diversity to the physicochemical properties of seawater. We found that the diversity and richness of microflora on the surface of microplastics with different quantity and time varied significantly. Proteobacteria are the main bacteria on microplastics, and the KEGG metabolic pathway prediction shows that amino acid metabolism and carbohydrate metabolism were abundant. In addition, there was a correlation between bacterial communities and Antibiotic Resistance Ontology (ARO). We used scanning electron microscopy (SEM) and Fourier transform infrared spectroscopy (FT-IR) techniques to evaluate the plastic polymer characteristics of disposable medical masks. Our research shows that disposable medical masks immersed in seawater can alter the microbial community. This study provides the most recent data and insights into the contamination of discarded masks in the marine environment.

## 1. Introduction

As the COVID-19 spreads worldwide, we human beings face serious health threats; in order to avoid the human nature transmission of coronavirus, people should wear a mask for protecting from the viral infection [1]. The global demand for masks wearing led to a significant increase in mask production [2]. The World Health Organization (WHO) predicts that the COVID-19 pandemic will continue for at least several years; therefore, the use of masks will also continue for several years. This has led to an increase in discarded masks [3]. During the COVID-19 pandemic, 129 billion masks were used globally each month [4], and about 3.5 million tons of masks were discarded worldwide without being treated. Many disposable masks have been found in the ocean [5]; a study reported that around 0.15 to 0.39 million tons of plastic can pollute marine ecosystems within a year due to improper waste management [6,7]. According to the Marine Conservation Organization report, in 2020, about 52 billion masks were produced worldwide, of which 1.56 billion entered the ocean through natural or artificial action, increasing the Marine ecosystem risk [8]. The increase in discarded masks is recognized as a new source of contamination associated with the COVID-19 pandemic [9,10]. Therefore, discarded masks may have an impact on microorganisms in seawater and thus alter the structure of microbial communities in the ocean [11].

The materials used in various face masks on the market contain polymer components. Usually made from non-degradable polymers such as polypropylene, polyethylene, polyester, polyacrylonitrile, and polyurethane [12,13], one of the materials most commonly used in mask production is polypropylene [14]. The face masks consist of three main layers: the outer layer is made up of polyester blend fabric with waterproof properties, the middle layer is composed of melt-blown fabric, and the inner layer consists of soft fiber [15]. Due to the special structural characteristics of the polypropylene material, the layers of most disposable medical masks are prone to release microfibers [16]. A study by Bussan et al. found that face masks not only contain trace elements but also release microplastics and emit trace elements [17]. The face masks also contain many toxic chemicals [18], such as polycyclic aromatic hydrocarbons (PAHs), bisphenol A, chlorinated phenols, polybrominated diphenyl ethers, nonylphenols, triclosan, and phthalates [19], which are harmful to all living things in the ocean by releasing a variety of toxic substances [20].

Face masks can release microplastics due to photodegradation, bio-corrosion, and immersion in water, progressively breaking smaller fragments over time, which have been identified as plastic pollutants in the marine environment [21,22]. These microplastics do not degrade easily and could exist in the marine environment for hundreds of years [23,24]. It is estimated that in coastal ecosystem environments, masks can release about 173,000 microplastic fibers per day [25]. These discarded masks various affect marine microorganisms in seawater in the form of microplastics. In addition, after decomposing into particles, discarded masks tend to adsorb organic and inorganic pollutants in the aqueous environment due to their small size, and affecting the toxicity of microplastics [26,27]. The mechanism of toxicity of microplastics is closely related to their physical properties and the contaminants in seawater. These pollutants accumulate on the surface of aquatic and may pose a risk of virus transmission, transport through rivers to the ocean, become microbial habitats, and affect environmental processes in marine ecosystems, resulting in marine ecological pollution.

The interaction between microplastics and microorganisms in seawater changes the structure of the microbial community to a large extent [28]. In addition, the physical and chemical properties of the masks are altered in the marine environment so that they can rapidly collect microorganisms and form plastic spheres with microbial communities in the form of biofilms [29,30,31,32,33]. The formation of biofilms on plastic surfaces promotes the adsorption of toxic pollutants and the growth of harmful microorganisms. The enrichment of harmful microorganisms on the surface of microplastics accelerates the adverse marine ecological effects [34]. Under the complex environmental conditions of marine ecosystems, microbial diversity and structure are influenced by a variety of factors: microplastic concentration, time of day, salinity, nutrients in seawater, and free-living bacteria all have important ecological effects on microbial community structure [35,36]. Currently, little is known about the marine ecotoxicity caused by disposable medical masks, the role of microorganisms in seawater, and their effects need further study [37].

The ecological impact of microplastics is considered to be an important field of research in marine ecology. A growing number of studies have reported that face masks are an emerging source of microplastic contaminants in the marine environment, and the massive use exacerbated an already dire situation. The accumulation of these contaminants on the surface of water bodies poses a risk of virus transmission and transport through rivers to the ocean, causing marine ecological pollution. To our knowledge, there are no data on the effects of microplastics from disposable medical masks on offshore microorganisms currently exists.

Little is known about the ecological risks posed by discarded masks in the marine environment. We hypothesized that the number of discarded masks immersed in seawater, their duration, and environmental factors would have an impact on the microbial community in seawater, and we conducted an experimental study to address this issue. We evaluated three time periods, 20, 40 and 60 days, to elucidate the correlation between the number of masks, immersion time, environmental factors and marine microorganisms through simulated experimental studies. To investigate the effects of masks on microbial diversity and community structure, and to explore the effects of microplastics on the metabolic functions and resistance genes of microbial communities in seawater. The microplastics were characterized by scanning electron microscopy (SEM) and Fourier transform infrared spectroscopy (FT-IR). Based on 16S rRNA gene sequencing and metagenomic sequencing, the structure and function of microbial communities and their ecological risks were studied.

## 2. Materials and Methods

### 2.1. Sample Collection

**Figure 1:**
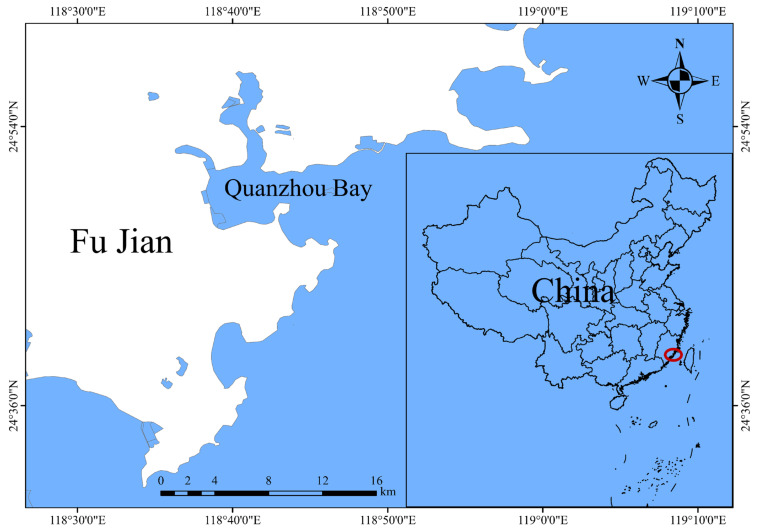
Map of sampling locations in the Quanzhou Bay of Quanzhou (Fujian Province, China).

Seawater was collected from the inlet of Quanzhou Bay, Quanzhou City (N: 24°51′47.50″, E: 118°47′33.62″), Fujian Province, China (Figure 1), and packaged back to the laboratory. All of the disposable medical masks were purchased from Youhekang (Guangzhou, China); they all complied with our medical device implementation standards, and were new and intact. Nylon filter membranes with 0.22 μm and 0.45 μm pore sizes were ordered from Haiyan New Oriental Plastic & Chemical Technology Co., Ltd. (Haiyan New Oriental Plastics Technology Co., Ltd., Jiaxing, Zhejiang, China). All chemicals used were of analytical grade and were used as received without any treatment.

### 2.2. Experimental Steps

In the laboratory, the experiments were conducted at room temperature; the collected seawater was divided equally into 12 glass bottles with a capacity of 10 L and divided into a total of four groups (C, L, M, and H) with three parallel groups each, and then each group was immersed with 0, 5, 25 and 50 disposable medical masks, where group C was a blank group without masks. We measured the parameters of environment for each sample at each time point (0 days, 20 days, 40 days and 60 days); the seawater physicochemical factors of (temperature (T), salinity (SAL), low dissolved oxygen (LDO), conductivity (CON), total dissolved solids (TDS), and pH were measured using HQ40D Portable Multi Meter (HACH Company, Loveland, CO, USA). A total of 1 L of seawater was collected at intervals (20, 40, and 60 days) for each sample, and we used Water-circulation multifunction vacuum pump (Zhengzhou, China) through a nylon filter membrane with 0.22 μm to obtaining planktonic microorganisms in seawater from all samples. The extracted samples were immediately stored in a refrigerator at −20 °C for subsequent DNA extraction and high-throughput sequencing analysis.

Microplastic sorting was performed by vacuum filtration through a 0.45 μm × 50 mm filter membrane to collect microplastics from four groups of seawater. To eliminate organic matter, samples are usually treated with a 30% hydrogen peroxide solution [38]. The material on the filter film was collected in a 500 mL beaker, and about 100 mL of 30% hydrogen peroxide solution was added, then sealed with foil; the solution was placed in a constant temperature oscillator (50 °C, 80 rpm) for 72 h to remove excess organic impurities in the sample. The digested solution was filtered again, and the filtered filter film was placed in a clean glass dish for drying and stored in a 4 °C environment for subsequent identification and analysis.

A sample of a brand-new disposable medical mask with outer, middle, and inner layers of material was cut with scissors into small pieces of less than 1 mm × 1 mm by sterile scissors and placed in a mortar and pestle to grind into fine particulate matter. The finished sample was placed in a dry sterilized glass Petri dish and covered with a surface dish for preservation for microscopic examination.

### 2.3. Quality Control

The ultrapure water used during the experiment was prepared from the milli-Q water purification system, and all glassware was washed three times with ultrapure water. To avoid contamination, particle-free nitrile gloves and laboratory cotton clothing were worn during the experiments, the laboratory table was scrubbed with 75% alcohol, all material (petri dish, forceps, filter) was sterilized, and the glassware used for the experiments was rinsed with ultrapure water before use and the filtration process was carried out in an ultraclean environment.

### 2.4. Sample Characteristics

We use reliable methods, techniques, and instruments for the analysis of microplastics, including morphological analysis (micromorphology) and chemical composition analysis (polymer composition). After sample processing, the three-layer mask fragments, as well as the solids on the filter membrane, require identification. Microplastics were characterized using scanning electron microscopy (SEM) with 2 kV electron accelerating voltage, to observe the microscopic form of matter. Fourier transform infrared spectroscopy (FT-IR) was used to identify the chemical composition of microplastics. Spectra were acquired in wavenumber ranges of 4000-400 cm^−1^ with 32 scans. The spectrum was obtained for each sample. All of the spectra were processed offline under the automatic baseline correction mode in the OMNIC software. By comparing the spectra of the samples with those of known materials, the types of polymers that make up the microplastics can be determined.

### 2.5. 16S rRNA Gene Sequencing

The planktonic bacterial communities in seawater from the blank and treated groups were collected by 0.22µm filters and DNA was extracted using CTAB/SDS method. DNA concentration and purity were monitored on 1% agarose gels. According to the concentration, DNA was diluted to 1 ug/μL using sterile water, the genomic DNA was used as the template. Amplify the V4 region of the 16S rRNA gene using a specific primer set (515F: 5′-GTGCCAGCMGCCGCGGTAA-3′ and the reverse primer 806R:5′-GGACTACHVGGGTWTCTAAT-3′). PCR reactions were carried out with 15 μL of Phusion^®®^ High-Fidelity PCR Master Mix (New England Biolabs); about 10 ng template DNA, and 0.2 μm of forward and reverse primers. Thermal cycling consisted of initial denaturation at 98 °C for 1 min, followed by 30 cycles of denaturation at 98 °C for 10 s, annealing at 50 °C for the 30 s, and elongation at 72 °C for 30 s, with a final extension of 72 °C for 5 min.

We mixed the same volume of IX loading buffer (contained SYB green) with PCR products and operate electrophoresis on 2% agarose gel for detection. PCR products were mixed in equidensity ratio. Then, the mixture PCR products were purified with Qiagen Gel Extraction Kit (Qiagen, Düsseldorf, Germany).

Sequencing libraries were generated using TruSeq^®®^ DNA PCR-Free Sample Preparation Kit (Illumina, San Diego, CA, USA) following the manufacturer’s recommendations and index codes were added. The library quality was assessed on the Qubit@2.0 Fluorometer (Thermo Scientific, Wilmington, DE, USA) and Agilent Bioanalyzer 2100 system. At last, the library was sequenced on an Illumina Hiseq platform by Novogene Co., Ltd. (Tianjin, China) and 250 bp paired-end reads were generated.

### 2.6. Statistical Analysis

A post-hoc test between different samples was performed in SPSS 26.0.0 software, and one-way ANOVA was used to evaluate the significant differences between samples, with a *p*-value of <0.05 regarded as statistically significant. Origin 9.90.225 was employed for plotting the species abundance map. The graphical representations of the results of environmental factors were performed using GraphPad Prism 8.0.2.263 for Windows.

## 3. Results and Discussion

### 3.1. Scanning Electron Microscopy (SEM) Analysis

**Figure 2:**
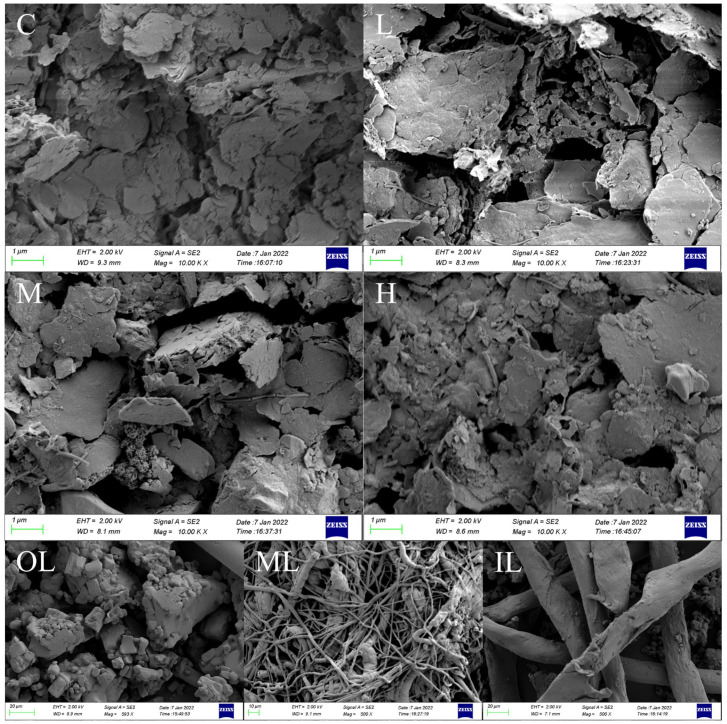
C, L, M, and H are the scanning electron microscopy images of microplastic particles in the control and experimental group collected samples, respectively; OL, ML, and IL are the SEM images of the outer, middle, and inner layers of the disposable medical mask, respectively.

To investigate the surface properties of microplastics, SEM was used to observe the microscopic changes in microplastic fibers in different groups. As can be seen from the SEM images (Figure 2), most of the microplastics have rough or even cracked surfaces. Blank Group C is microplastics in pristine seawater, which are more severely weathered, probably because they have been present in seawater for a longer period. New microplastics were apparent in groups L, M, and H. We speculate that the microplastics were released from the masks immersed in seawater. The fragments in group L were larger and showed surface damage. The microplastics in group M showed some fibrous fragments and small particles. The microplastic surface of the fiber became abrasive in group H. The pictures show that the more the masks are immersed in seawater, the more visible the granularity of microplastics becomes. Microplastics of different shapes were obtained from the three layers of the mask with fibers resulting from grinding of the inner, middle, and outer layers. The outer layer produced irregularly shaped pieces, the inner layer consisted of a grid of fibers of uniform diameter, and the middle layer had significantly thinner fibers with a tighter mesh structure and intertwined, probably because the composition of the disposable medical mask material was different between the layers.

### 3.2. Fourier Transform Infrared Spectrometer (FT-IR) Analysis

**Figure 3:**
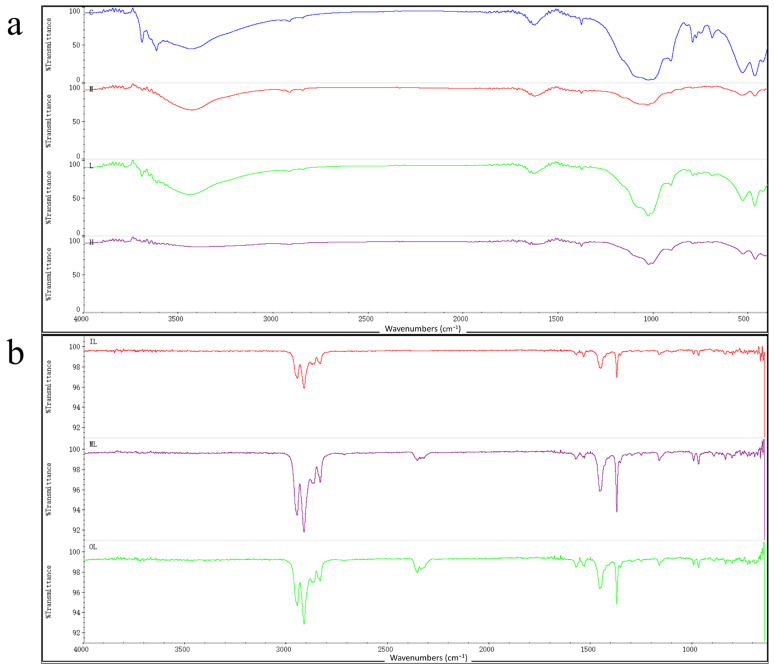
(**a**) C, L, M, and H are the FT-IR spectra of each group of collected samples, respectively; (**b**) OL, ML, and IL are the infrared spectra of the outer, middle, and inner layers of disposable medical mask, respectively.

Figure 3a shows the FT-IR spectra of the solid on the filter membrane, and the FT-IR spectra of all four sets of samples show characteristic bands similar to those of the polymer. A comparison of the sample spectra with the polymer spectra in the Hummel Polymer Sample Library revealed less similarity with polypropylene and polyethylene in the experimental group, but not in the blank group. It can be shown that masks immersed in seawater release microplastics containing polypropylene components. Long-term studies have shown that microplastics can cause marine ecotoxicity. Further research will be conducted on the effects of microplastics from disposable medical masks on offshore microorganisms.

The chemical structures of the three mask fragments as well as the solids on the filter membrane were analyzed by FT-IR analysis. Figure 3b shows the FT-IR spectra of the three-layer masks, where the peaks caused by the vibrations indicate that the peaks generated by the asymmetric vibrations of CH_2_ and CH_3_ are characteristic bands of the polypropylene crystal structure, including the small peaks appearing at 1200–800 cm^−1^. The FT-IR spectra of the outer, middle, and inner of the masks were compared with the polymer profiles from the Hummel Polymer Sample Library, and the positions and the with relative intensities of the absorption bands were identical to those of polypropylene; in addition, the target spectra were also found to be similar to those of polyethylene and other polymers, which confirmed that the disposable medical masks indeed contain polypropylene, polyethylene, and other polymers. FT-IR spectroscopic analysis of the intact masks showed that all three layers of the masks contained polypropylene components in their materials. Therefore, the large number of plastic particles contained in masks and their potential ecological risks may require extensive attention [39].

### 3.3. Analysis of Microbial Community Abundance and Diversity

To understand the effects of microplastics released from face masks on the microbial communities in seawater, a total of 3,073,341 high-throughput bacterial sequences were generated, ranging from 76,053 to 95,067 per sample and based on 16S rRNA gene sequencing. We assessed the richness and diversity of microorganisms in all of the samples, dividing all of the sample sequences into operational classification units (OTUs) according to the distance between the sequences, a total of 4007 OTUs were found after clustering all of the sequences based on 97% sequence similarity. The coverage index in all of the samples was more than 97%, indicating that the bacterial sequence in the sample was detected. The higher the Coverage value, the higher the probability of the sequence being detected in the sample, that is, the sequencing results could reflect the authenticity of the samples, and the dilution curve tended to flatten out, indicating that the sequencing data volume was large, and the samples were sequenced completely. We analyzed the species annotation results of OTUs, analyzing sample bacterial community complexity by alpha diversity, the impact of microplastics on bacterial alpha diversity may be the result of multiple factors, and the morphology of microplastics may also differentiate the evaluation results. In this study, the Chao1 and ACE indices are applied for evaluating bacterial community richness, while the Simpson and Shannon indices are used for evaluating bacterial community diversity (species richness and evenness).

Bacterial community complexity diversity was evaluated using indices including Simpson, Shannon, ACE, and Chao1, as shown in Table 1, the OTUs values were increased in the collected samples in the experimental group compared to the control group, ACE and Chao 1 indexes rose in experimental groups L, M, and H, indicating that microplastics released from disposable medical masks promoted microbe richness, with a maximum value in group L, indicating that the bacterial community at this time was richer than the other experimental groups.

Shannon diversity estimates varied between 4.890–5.446, with higher Shannon values indicating higher community diversity, compared with the control group, the Shannon index increased in the experimental group, especially with the largest change in the experimental group H with 50 disposable medical masks. The results of this study showed that the species richness of bacteria in C was significantly lower than L, M, and H (ANOVA, *p* <0.05), while neither the Shannon index nor Simpson index showed significant differences (ANOVA, *p* > 0.05), indicating that there was no significant difference in species richness in groups L, M, and H.

**Figure 4:**
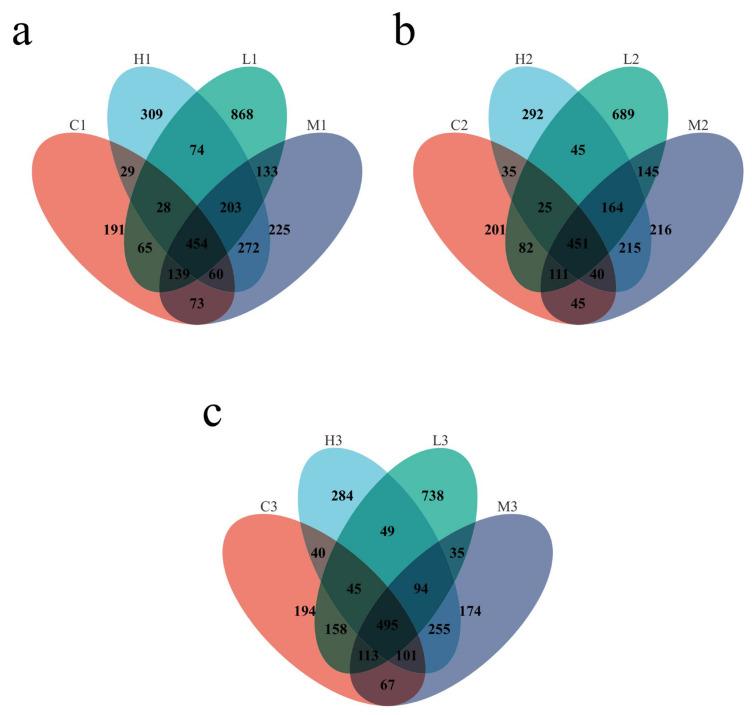
(**a**) Venn diagram between groups at 20 days; (**b**) Venn diagram between groups at 40 days; (**c**) Venn diagram between groups at 60 days.

As shown in Figure 4, the Venn plot shows the shared and specific OTUs in the different groups. The dominant microbiotas existing in the four groups were studied. The figure shows bacterial OTUs values on microplastic surfaces at 20d, 40d, and 60d, respectively; in total, 454, 451, and 495 OTUs were shared among the four groups in three time periods. In the later stages of the experiment, the composition of the bacteria was enriched, indicating some differences in bacterial communities on microplastic surfaces at different times. In addition, the bacterial community structure attached to microplastic surfaces varied over time, with unique bacterial OTUs in each sample, with 191, 868, 225, and 309 at 20d, 201, 689, 216 and 292 at 40d and 194, 738, 174 and 284 at 60d.

There was no significant difference in the Alpha diversity of bacterial communities among the four groups in the initial stage of the experiment, while the Alpha diversity index of the experimental group was higher than that of the water samples in the middle and later stages of the experimental exposure, and the ACE, Simpson, and Shannon indices all showed an increasing trend, which may be due to the complex interaction between microplastics and microorganisms that changed the bacterial community structure, indicating that the masks may provide an environment for microorganisms in seawater to survive and reproduce. In addition, the microbial diversity of the samples in the experimental group increased with prolonged exposure to seawater and the composition of the microbial community changed significantly with time, indicating multiple effects on the diversity of the bacterial community attached to the surface of the masks in seawater.

### 3.4. Analysis of the Microbial Community Structure

**Figure 5:**
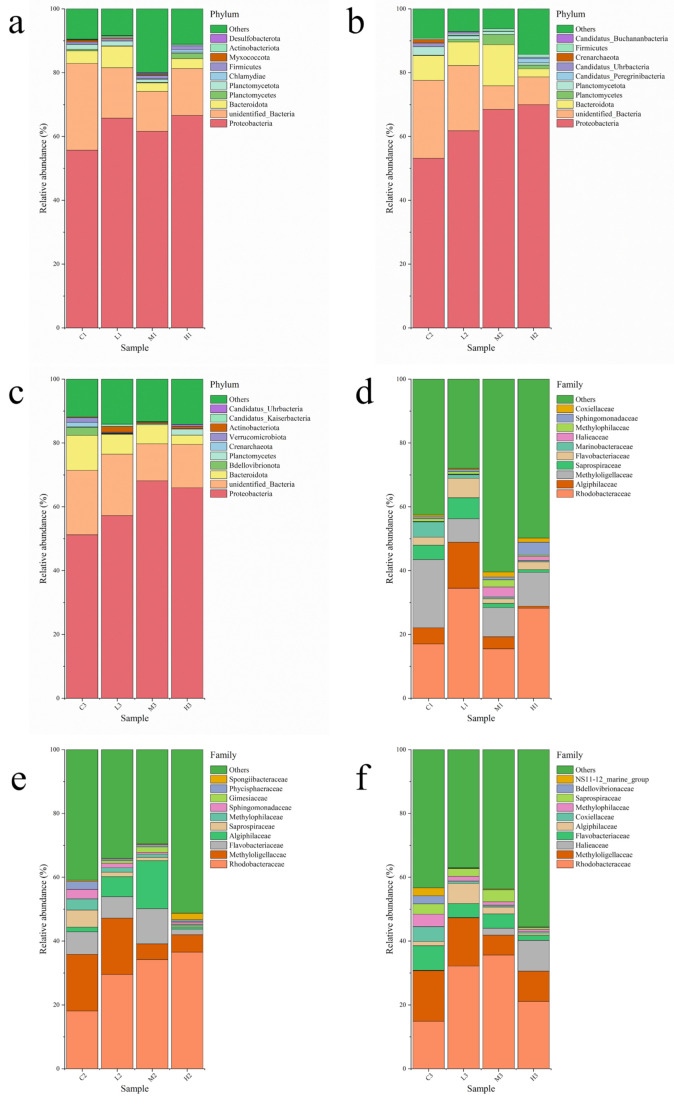
(**a**–**c**) Microbial community abundance maps at the phylum level; (**d**–**f**) Microbial community abundance maps at the family level.

A total of 51 phyla, 97 orders, 214 orders, 300 families, 517 genera, and 211 species of bacteria were detected in this study. For the bacterial community composition at the phylum level, different periods and different numbers of masks have different effects. The dominant phyla were identical for each sample, but the relative abundance of each species changed after the addition of masks, indicating that the time and number of mask immersion had effects on species abundance. Figure 5a–c shows the bacterial community structure at the level of control and experimental groups, with the top ten bacterial phyla in seawater richness representing 78.16% to 93.15% of all sequence reads, and the remaining bacterial phyla merged as “Other”. Proteobacteria (51.20–69.99%) is the dominant strain in all collected samples, the second most abundant bacterial phylum was unclassified and was marked by unidentified_Bacteria (7.43–27.21%), then came Bacteroidota (2.52–12.87%) and Planctomycetes (0.094–3.21%). At 40d and 60d, the relative abundance of Proteobacteria in all collected samples in L, M, and H increased compared with the control C samples, indicating that the microplastics released by masks soaked in seawater promoted the growth of Proteobacteria. The relative abundance of Bacteroidota in all collected samples in M and H was significantly lower than that of the control C samples.

Figure 5d–f shows the bacterial community structure at the family level for the control and experimental groups, showing the top ten bacterial genera in abundance and merging the other species into “others”. At the family level, the top 10 families included Rhodobacteraceae, Algiphilaceae, Methyloligellaceae, Saprospiraceae, Flavobacteriaceae, Marinobacteraceae, Halieaceae, Methylophilaceae, Sphingomonadaceae, and Coxiellaceae. Of them, Rhodobacteraceae, Methyloligellaceae, Algiphilaceae, Flavobacteriaceaen, and Saprospiraceae, have been identified as the abundant families in all samples. The low relative abundance of Gimesiaceae, Phycisphaeraceae, and Spongiibacteraceae appeared at 40d and Bdellovibrionaceae, NS11-12_marine_group appearing at 60d. The relative abundance of Spongiibacteraceae increased with the number of masks, and the relative abundance of Phycisphaeraceae, Bdellovibrionaceae, NS11-12_marine_group decreased with the number of masks. Coxiellaceae and Halieaceae appear at 20d and 60d. Coxiellaceae and Halieaceae appear only at 20d and 60d. However, the four groups differed in the structure of the bacterial community at each period. The results showed that the relative abundance of Flavobacteriaceae, Methyloligellaceae, Halieaceae, Methylophilaceae, and Coxiellaceae increased and that of Algiphilaceae and Saprospiraceae decreased with increasing time. These results suggest that the seawater environment immersed with masks is conducive to the growth of some microorganisms and provides a favorable living environment for marine microorganisms.

In this study, there was a significant difference in bacterial community richness between all groups in which Proteobacteria were predominant, Bacteroidetes and unclassified phylum were secondary. The richness of the bacteria changed significantly during the late stages of the experiment, with proteobacteria increasing and Bacteroidetes decreasing. The previous finding that Bacteroidota could degrade polymer compounds also showed that masks could affect the abundance of Bacteroidetes in the later stages of the experiment. The relative abundance of Methyloligellaceae, Flavobacteriaceae, and Methylophilaceae increased, indicating that these bacteria readily adsorb on mask particles to grow and reproduce.

### 3.5. Clustering and Correlation Analysis of Microorganisms and Environmental Factors

**Figure 6:**
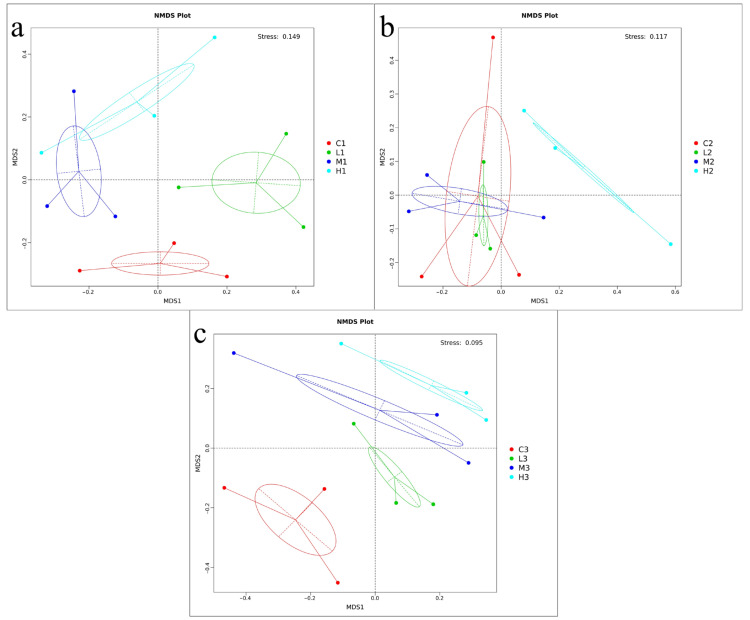
Comparison of bacterial community diversity across different periods and between different samples, with NMDS based on Bray−Curtis similarities (stress values are less than 0.2); (**a**) MNDS profiles of bacteria between samples at 20d; (**b**) MNDS profiles of bacteria between samples at 40d; (**c**) MNDS profiles of bacteria between samples at 60d.

NMDS analysis based on the Bray-Curtis similarity of OTUs in mask-released microplastic samples analyzed the microbial community dynamics of seawater and different numbers of masks on the temporal scales, and the differences in bacterial communities among the four groups in more detail. As depicted in Figure 6, the bacteria were significantly separated in the bacterial community structures between the three-time points on the temporal scale, and the three samples in each group gathered together with high reproducibility, indicating statistically significant differences in the microbial diversity between groups. In Figure 6, it can be seen that in the initial stage (20d) and final stage (60d), the samples in each group were clustered together, indicating that differences were significantly greater than within groups and significant in bacterial community diversity. At 40d, the clustering in each group was not obvious; when the confidence ellipses of the microplastic-associated bacterial communities of the L and M samples overlap, this indicated the similarity between the bacterial communities in the two groups. The bacterial communities on group H samples were relatively separated from the other samples, indicating the low similarity between samples.

### 3.6. Correlation Analysis of Microbial Diversity and Environmental Factors

**Figure 7:**
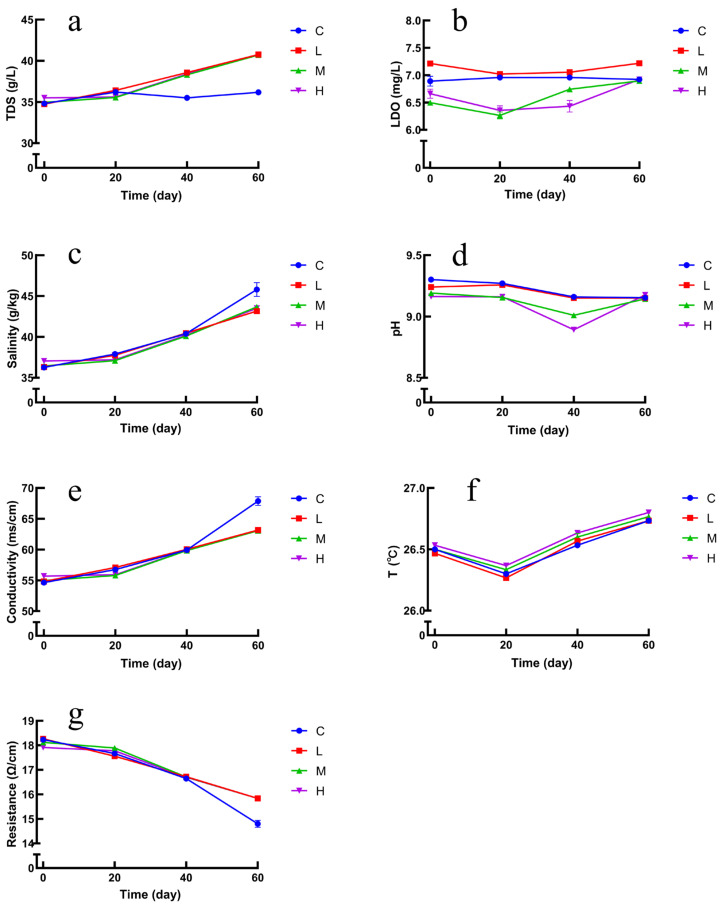
Effects of total dissolved solids (**a**), dissolved oxygen (**b**), salinity (**c**), pH (**d**), conductivity (**e**), temperature (**f**), and resistance (**g**) in seawater.

**Figure 8:**
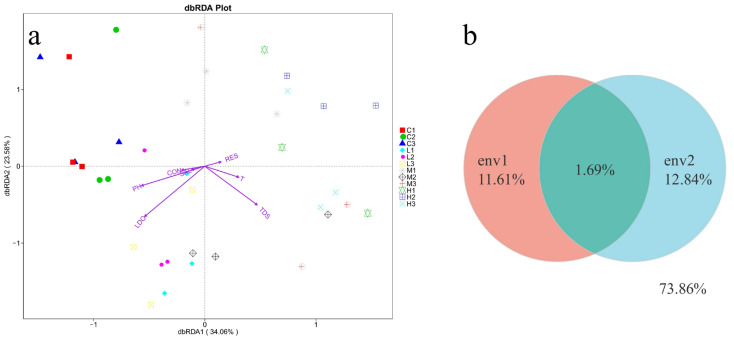
(**a**) Redundancy analysis (RDA) analyzed the correlation between bacterial Alpha diversity and environmental factors (temperature, salinity, dissolved oxygen, pH, etc.), each dot in the figure represents a single sample, and samples from the same group are represented using the same color, the direction indicated by the arrow indicates the trend of environmental factors with length proportional to correlation. (**b**) The circle overlap indicates the common amount of interpretation shared between the first set of environmental factors and the second group, and the circle indicates the uninterpretable nature.

In the present study, the trends of environmental factors are shown in Figure 7a–g. At 20d, 40d, and 60d, TDS, salinity, and conductivity increased in the experimental group compared with the blank group, where the increase in TDS indicates that some kind of substance has increased in the seawater, further indicating that it may be that the masks released microplastics in the seawater and increased with time, and the changes in resistance showed a decreasing trend. The changes in LDO, pH, and temperature were not significant. The correlation between bacterial composition and environmental factors was explored by redundancy analysis (RDA). RDA analysis was performed on all samples to assess the correlation of bacterial diversity with environmental factors (temperature, salinity, pH, dissolved oxygen, total dissolved solids, etc.). Combining the RDA results, LDO, TDS, and pH were the factors that influenced the diversity of bacterial communities between the groups. Figure 8a illustrates that the growth of bacteria was positively correlated with LDO and pH. Bacterial community diversity was less correlated with salinity, temperature, and electrical conductivity. The RDA results illustrated that environmental parameters significantly differentiated the bacterial community, and all samples displayed a gradual change in environmental parameters such as LDO, salinity, and pH. In addition, further evaluation of environmental factors on the bacterial distribution using VPA analysis, as shown in Figure 8b. The changes in bacterial community structure were observed in the OTUs, with 11.61% of salinity, low dissolved oxygen, total dissolved solids, and 12.84% of temperature, pH, conductivity, and resistance. The amount of explanation shared by these two sets of environmental factors was 1.69%. In the OTUs data, 73.85% of the number of changes in bacterial distribution could not be explained by environmental factors, indicating that other factors also affect the microbial community.

Environmental factors such as temperature, salinity, pH, and low dissolved oxygen impact the diversity and relative abundance of bacterial structures in seawater immersed with different numbers of masks. The total dissolved solids varied across stages, possibly due to increased microplastics released by masks over time. Some studies found that salinity was the main factor affecting the diversity of microplastic bacterial communities [40], while the present study found that salinity was not a major effect.

### 3.7. Mask Exposure Resulted in Significant Changes in the Predicted Seawater Microbial Function

**Figure 9:**
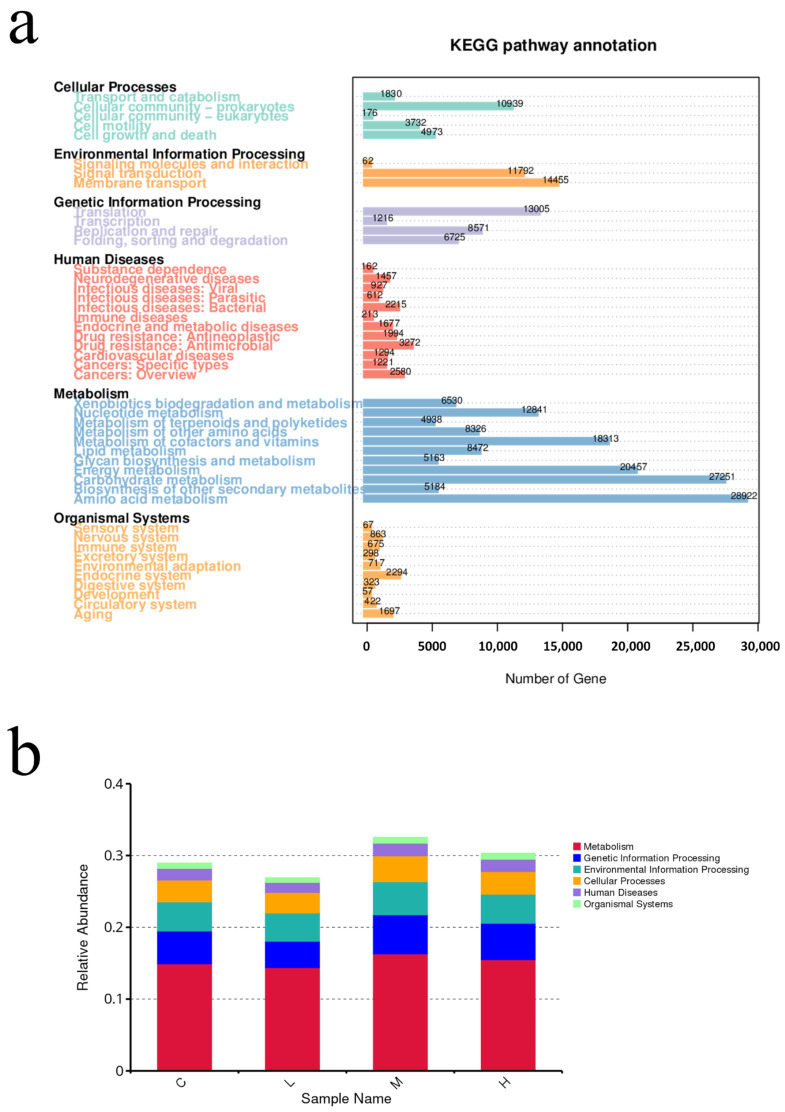
(**a**) Functional prediction of the KEGG metabolic pathway. (**b**) Histogram of relative abundance of functional annotations on level1.

The metagenomic sequencing profiling data of samples were annotated to the KEGG database to evaluate the functional diversity. Figure 9a shows the differences in the potential functional abundance of the bacterial community between the groups. The results of the pathway annotation showed that the metabolic pathways were different in each group. The most enriched pathways were concentrated in metabolism related pathways (58.82%), followed by genetic information processing related pathways (11.86%), environmental information processing related pathways (10.57%), and cellular processes related pathways (8.70%). In microbial survival, the main pathways with the highest enrichment of basic metabolic function genes are amino acid metabolism (11.62%), carbohydrate metabolism (10.95%), energy metabolism (8.22%), metabolism of cofactors and vitamins (7.36%), membrane transport (5.81%), translation (5.22%), nucleotide metabolism (5.12%), signal transduction (4.74%), cellular community-prokaryotes (4.39%) and other high proportion of microbial functional genes, the proportion of total enriched genes in each pathway was above 4.0%. In the middle and later stages of the experiment, the function of seawater microorganisms changed. By comparing the relative abundance of functional genes in different experimental groups, it was found that the experimental group had a significant increase in metabolism, genetic information processing, and environmental information processing (Figure 9b).

The KEGG function annotated results showed that some metabolic pathways were enriched in microplastic related communities, such as functional genes related to microbial metabolism (amino acid metabolism, carbohydrate metabolism) higher abundance, abundant metabolic function types related to the growth and reproduction of bacteria, indicating that masks in seawater affect the potential function of microbial communities, thus indirectly affecting the ecological function of microbial communities in the Marine ecosystem.

### 3.8. Effect of Microplastics on Resistance Genes

**Figure 10:**
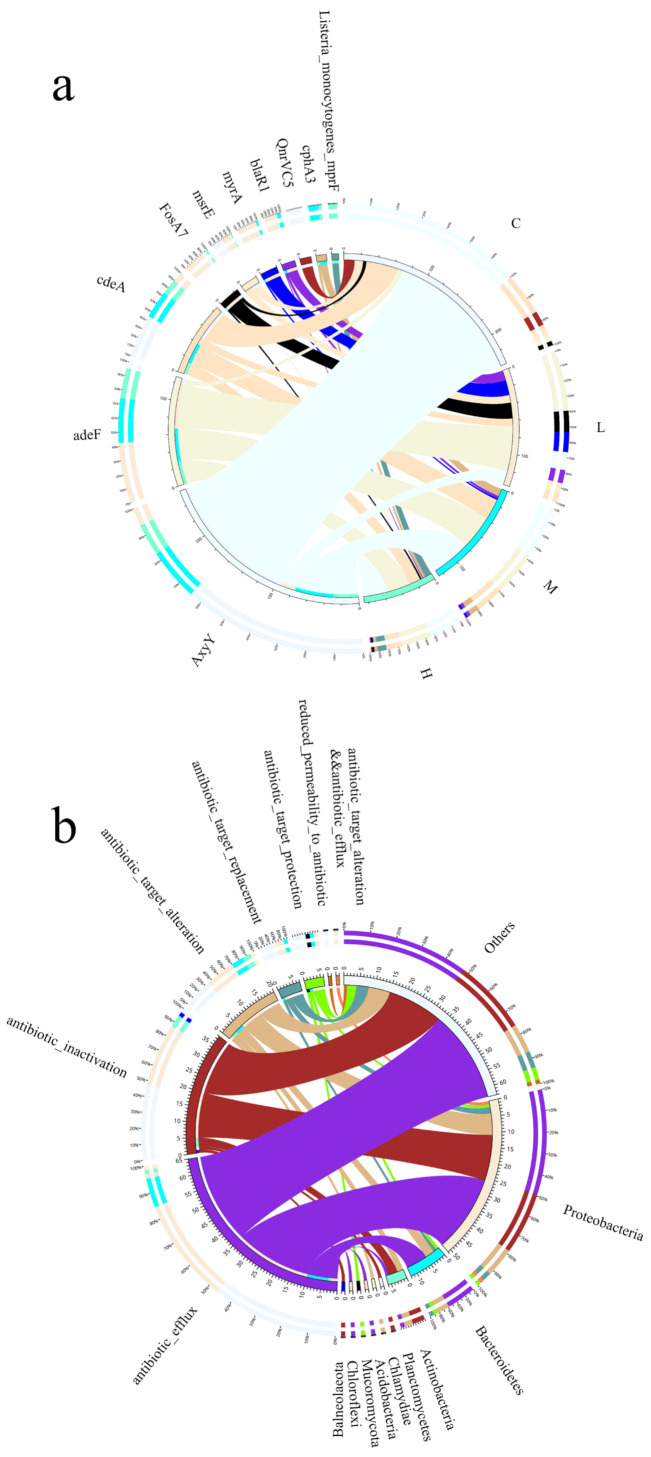
(**a**) Resistance genes overview circos diagram, sample information on the right and ARO information on the left. (**b**) Resistance mechanisms and species overview circos diagram, information on phylum level species is shown on the right, and information on resistance mechanisms is shown on the left.

Due to their complex surface structure, microplastics tend to adsorb contaminants from seawater, such as antibiotics, which directly affects the properties of microplastics and the structure of the microbial community in seawater. In this study, the abundance of seawater resistance genes differed between the four groups (Figure 10a), with the addition of masks reducing the abundance of seawater resistance genes in the group compared to the blank control, indicating that microplastics interfered with seawater microbial processes and affected the abundance of seawater antibiotic resistance genes. The dominant microbial communities at the phylum level were classified into Proteobacteria, Bacteroidetes, Actinobacteria, Planctomycetes, Chlamydiae, and Acidobacteria showed in Figure 10b, Antibiotic_efflux, Antibiotic_inactivation, Antibiotic_target_alteration, the resistance genes in each resistance mechanism accounted for 49.02%, 29.41%, and 13.73% of the resistance genes in Proteobacteria, respectively, and accounted for 47.62%, 28.57%, and 11.11% of the resistance genes in Others, respectively. Microbial communities on microplastics are a potential risk in ARO dissemination. The persistence and widespread distribution of microplastics in seawater further accelerate the accumulation of AROs in the marine environment as they continue to drift through the ocean, making the marine ecosystem even more polluted.

The results show that microplastics have an effect on the abundance of resistance genes and that the mechanism of action of resistance genes is also related to microorganisms in seawater [41], suggesting that the combined contamination of microplastics and resistance genes may cause marine ecological risks [42]. Contamination from discarded masks can be long term and we should therefore be aware of the risks posed to marine ecosystems by ARO contamination associated with face masks [43].

## 4. Conclusions

In this study, we investigated the effects of discarded masks on the structure and function of offshore microorganisms. We found that the masks contained polypropylene components and that bacterial diversity and community structure changed significantly in seawater immersed with the masks. The relative abundance of bacterial communities in each sample varied significantly in the middle and later stages of the experiment, especially for Proteobacteria and Bacteroidota. TDS and LDO are important environmental factors affecting bacterial diversity. Our results further indicate that KEGG functional gene predictions show that amino acid metabolism and carbohydrate metabolism are more abundant, there is a correlation between bacterial community structure and ARO, and the delivery mechanism of ARO can disrupt the microbial balance in the marine environment and trigger ecological risks. Although the limitation of this study is that the environmental conditions offshore cannot be fully simulated, the information and data obtained can help to assess the impact of discarded masks on ecological processes in the structure of the offshore microbial community.

## Figures and Tables

**Table 1:** Sequence information and diversity index of microbial diversity.

Group	OTU	ACE	Chao1	Shannon	Simpson	Coverage
C	615 ± 59.95 b	713.36 ± 91.39 c	694.42 ± 88.05 c	4.890 ± 0.275 a	0.894 ± 0.0331 a	0.99
L	906 ± 44.73 a	1035.62 ± 55.57 a	1011.84 ± 53.11 a	5.216 ± 0.120 a	0.914 ± 0.0156 a	0.99
M	802 ± 63.25 a	903.63 ± 58.33 b	881.64 ± 56.95 b	5.200 ± 0.399 a	0.909 ± 0.0266 a	0.99
H	827 ± 35.29 a	930.33 ± 39.49 ab	909.98 ± 35.92 ab	5.446 ± 0.227 a	0.913 ± 0.0253 a	0.99

The larger Shannon and Simpson indices indicate the higher microbial community diversity in the sample; the larger Ace and Chao1 indices indicate the higher microbial community richness; different lowercase letters (a, b, c) indicate significant levels of difference consistent with one-way ANOVA and Tukey’s post-hoc test results (*p* < 0.05).

## Data Availability

Not applicable.
